# Reduction of Hexavalent Chromium by Stenotrophomonas and Bacillus

**DOI:** 10.1002/mbo3.70286

**Published:** 2026-04-09

**Authors:** Ahmad Fadhlullah Husaini, Margaretta Christita, Rizal Maarif Rukmana, Arida Susilowati, Keni Vidilaseris

**Affiliations:** ^1^ Research Center for Applied Microbiology, Research Organization of Life Sciences and Environment, National Research and Innovation Agency (BRIN) Cibinong Science Center‐BRIN Complex Bogor Indonesia; ^2^ Department of Biochemistry, Faculty of Mathematics and Natural Sciences IPB University Bogor Indonesia; ^3^ Research Center for Pharmaceutical Ingredients and Traditional Medicine, Research Organization for Health, National Research and Innovation Agency (BRIN) Cibinong Science Center‐BRIN Complex Bogor Indonesia; ^4^ Faculty of Forestry Universitas Sumatera Utara Deli Serdang North Sumatra Indonesia; ^5^ Department of Molecular and Integrative Biosciences, Faculty of Biological and Environmental Sciences University of Helsinki Helsinki Finland

**Keywords:** bioremediation, chromate reductase, environmental microbiology, heavy metal resistance, hexavalent chromium, microbial ecology

## Abstract

Hexavalent chromium [Cr(VI)] is a widespread environmental pollutant, posing a significant health risk to ecosystems and humans. Bioremediation using microorganisms offers a cost‐effective strategy for its detoxification. This review highlights recent advances in Cr(VI) reduction by *Stenotrophomonas* and *Bacillus* species, two bacterial genera with strong potential for chromium detoxification. *Stenotrophomonas* species primarily rely on intracellular enzymatic reduction mechanisms, often mediated by chromate reductases such as ChrR and heme proteins that link chromium detoxification with iron homeostasis. In contrast, *Bacillus* species employ a broader range of strategies, combining intracellular and extracellular enzymatic reduction, biosorption, and bioaccumulation, supported by stress‐response and efflux systems that confer exceptional tolerance to Cr(VI). Comparative analysis reveals complementary metabolic strengths: *Stenotrophomonas* excels in rapid enzymatic detoxification, while *Bacillus* offers long‐term stability through spore formation and surface‐associated sequestration. Together, these traits underscore the promise of mixed consortia featuring both genera for scalable and resilient chromium bioremediation systems. Future research integrating omics‐guided pathway mapping, microbial engineering, and biosafety control is expected to accelerate the deployment of efficient and safe Cr(VI) bioremediation technologies.

## Graphical abstract

Stenotrophomonas and Bacillus remove toxic hexavalent chromium via enzymatic reduction and non‐enzymatic mechanisms. Reductases (e.g., ChrR, HhuH, YhdA, NfrA, Bcr005, NitR, AzoR) use NAD(P)H to convert Cr(VI) to the less toxic Cr(III).

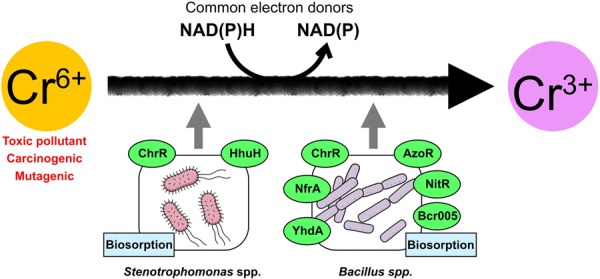

## 1 Introduction

Increasing levels of chromium contamination from industrial and mining waste, such as sludge, pose a serious environmental and public health threat in the surrounding areas. Hexavalent chromium [Cr(VI)], a stable and commonly used industrial form, is a highly toxic, water‐soluble, and mobile form of chromium widely used in industries such as leather tanning, electroplating, and steel manufacturing for its hardness, durability, and corrosion resistance (Saha et al. 2022). According to the EPA IRIS database (2004), Cr(VI) can damage the gastrointestinal tract, liver, reproductive organs, and lung, and is linked to immune, hematological, and male reproductive toxicities. Cr(VI) easily enters cells via sulfate channels, triggering oxidative damage, DNA mutations, chromosomal abnormalities, and cell‐cycle disruption (Wakeman et al. 2017). Human exposure via ingestion, inhalation, or skin contact (H. Ge et al. 2019; Nickens et al. 2010), especially in high‑risk occupations (Islam et al. 2022), can cause respiratory inflammation, skin ulcers, liver and kidney injury, and a significantly elevated risk of lung cancer (Gibb et al. 2020) with some studies reporting a 2–80‑fold increase (Holmes et al. 2008).

In the environment, chromium is abundant in the Earth crust and primarily occurs as Cr(III) in chromite minerals within ultramafic rocks (Stefánsson et al. 2015). However, under oxidizing conditions, often mediated by manganese oxides, Cr(III) can be converted into Cr(VI), which is more soluble and mobile, especially in neutral to alkaline pH (Aiken et al. 2023; Botsou et al. 2022). While Cr(VI) can be formed naturally, most environmental contamination stems from anthropogenic sources such as industrial emissions and waste (Chrysochoou et al. 2016). Once released, Cr(VI) can travel through air, soil, and groundwater, with its persistence and mobility influenced by redox potential, organic matter, and metal oxides. These properties make Cr(VI) a widespread and long‐lasting pollutant with significant ecological and public health implications, unless effectively remediated (Briffa et al. 2020).

While existing conventional physicochemical treatments such as precipitation (Ramakrishnaiah and Prathima 2011), ion exchange (Flint et al. 2021; Leonard et al. 2023), electrocoagulation (Genawi et al. 2020), and nanoparticle‐based adsorption (Brasili et al. 2020; Farooqi et al. 2021; Srivastava et al. 2016; Tolkou et al. 2020) can effectively immobilize or reduce Cr(VI) to the less toxic Cr(III), these methods often generate secondary waste (Malairajan et al. 2007; I. B. Singh and Singh 2001) and involve high operational cost, making them impractical for treating large‐scale contamination (Aigbe and Osibote 2020). Consequently, biological reduction of Cr(VI) using microorganisms, such as bacteria, has gained considerable attention as a sustainable, environmentally friendly, and cost‐effective alternative (J. Chen and Tian 2021).

Bacteria employ various mechanisms, including enzymatic reduction and biosorption processes, to transform Cr(VI) into stable, less toxic, and less mobile Cr(III) without generating secondary waste. Their natural abundance and natural resistance to heavy metal toxicity make them ideal candidates for large‐scale bioremediation. Among these, species from the genera *Stenotrophomonas* and *Bacillus* have demonstrated remarkable potential as bioremediation agents (Baldiris et al. 2018; Ramírez et al. 2019). Mukherjee and Roy (2016) have described the broad genetic potential in bioremediation encoded in *Stenotrophomonas* genomes due to the existence of several genes implicated in xenobiotic degradation, heavy metal resistance, and stress response. These genomic features might provide the basis for the genus adaptability and accounts for the frequent isolation from contaminated and industrial sites such as tannery industry in India (Sundari 2017), chrome plating wastewater in Colombia (Baldiris et al. 2018), and soils from industrial park in China (S. Ge et al. 2015). Chromium reduction capabilities in some isolates have been linked to the chromate reductase gene (*chrR*) and various efflux systems, which support both high tolerance and efficient reduction of Cr(VI) (Baldiris et al. 2018), with reduction rates varies depending on the strain and environmental conditions. Under optimized laboratory settings, *S. maltophilia* NA2 can achieve reduction rates as high as 6.39/mg/L/h (Baldiris et al. 2018). In stark contrast, under metabolic burden of co‐contaminants like PCBs, the reduction rate can drop as low as 0.019 mg/L/h in *S. maltophilia* K8 (Yasir et al. 2021), as will be discussed later in detail. This broad range of reduction rates due to diverse genetic makeup makes *Stenotrophomonas* an ideal subject for chromium bioremediation.

Alongside *Stenotrophomonas*, the genus *Bacillus* is also a popular subject in chromium bioremediation research, valued for both its industrial biotechnology utility and its exceptional resilience to environmental stressors. A key advantage of this genus is the ability to form highly resistant endospores that enable persistence in harsh environments where other microbes would not survive (McKenney et al. 2013). This resilience is reflected in extreme chromium tolerance observed in several isolates. While many effective strains tolerate several hundred mg/L of Cr(VI), *Bacillus* sp. MH778713 exhibits a remarkable Minimal Inhibitory Concentration (MIC) of 15.000 mg/L (Ramírez et al. 2019). This tolerance is frequently coupled with notably high detoxification efficiency, as demonstrated by strains such as *Bacillus paramycoides* Cr6, which exhibit rapid reduction rates of up to 11.11 mg/L/h and can completely remove 200 mg/L of Cr(VI) within 18 h (Gu et al. 2023). The adaptability of the *Bacillus* genus in chromium‐contaminated environments is further highlighted by its successful isolation from a wide range of sites, including tannery effluents (Elahi et al. 2022), chromite mine soils (Dhal et al. 2010), and extreme alkaline soda lakes (Ibrahim et al. 2012). The combination of innate resistance, fast reduction rates, and widespread environmental presence makes this genus a strong candidate for bioremediation of chromium‐contaminated sites.

Despite growing interest in bacterial chromium reduction, a comprehensive understanding of the capabilities, mechanisms, and optimal conditions for *Stenotrophomonas‐* and *Bacillus*‐mediated Cr(VI) reduction is still lacking for the development of effective remediation strategies. This review examines current knowledge for chromium reduction by these two genera, evaluates their potential for environmental application, and highlights key research gaps to advance microbial bioremediation and address the global challenge of chromium contamination.

## 2 Current Remediation Strategies for Hexavalent Chromium

Conventional Cr(VI) remediation employed a diverse physicochemical approach incorporating advanced materials and engineering techniques. Chemical‐based remediation is widely used, utilizing various reducing agents such as zero‐valent iron (Fang et al. 2021), ferrous sulfate heptahydrate (Mončeková et al. 2016), elemental sulfur (Sahinkaya et al. 2012), or sodium metabisulfite (Azis et al. 2021). New research has shed light on using a more green‐based chemical from plant extract, such as gallic acid (Mystrioti et al. 2021), Quercetin (Okello et al. 2012), and tannic acid (Jiang et al. 2022) as reducing agents for Cr(VI). Conventional methods for chemical reduction of Cr(VI) occur in two stages: acidification to convert Cr(VI) to Cr(III), followed by alkalinization to precipitate the Cr(III) hydroxide (Cho et al. 2023; Lei et al. 2023). Electroreduction combined with coagulation has also been intensively studied, using various electrode materials such as iron (Tochetto et al. 2024), aluminium (Tejada tovar et al. 2021), carbon paste (Hilali et al. 2018), graphite (P. Chen et al. 2021), and titanium (X. Zhang et al. 2023). This technique relies on electrolytic reduction at the cathodes and coagulation at the anodes, with system performance heavily influenced by electrode composition and spacing, factors that can increase deployment complexity and cost in a real‐world setting.

Bioremediation of Cr(VI) leverages plants, microbes, and fungi using mechanisms like phytoremediation, biosorption, bioaccumulation, and enzymatic biotransformation, bridging microbial biotechnology and environmental engineering (Jia et al. 2022; John and Rajan 2021; Nisa et al. 2025). Phytoremediation employs plant root systems and their associated microbes to absorb, filter, or immobilize Cr(VI) from soil or water, with species like *Pteris vittata* (Chinese brake fern) demonstrating significant pollutant uptake and translocation into aerial tissues (Jia et al. 2022; Sridhar et al. 2011). However, harvested biomass must be managed safely as it might still contain Cr(VI). Microbes in the rhizosphere, especially plant growth–promoting rhizobacteria, further enhance phytoremediation through Cr(VI) reduction, siderophore production, and stress alleviation (Jia et al. 2022).

Bacterial bioremediation operates via biosorption and bioaccumulation through active metal uptake, often outperforming traditional methods in cost and biodegradability (Ansari et al. 2011; Priyadarshanee and Das 2021). Bioaccumulation is the accumulation of various pollutants, including heavy metals, inside the cells of organisms from dietary sources and the environment, which involves the adsorption of heavy metals and active transport of the metal species into the cells (Nnaji et al. 2023). Some bacteria can transform heavy metals into less toxic or more soluble forms through biotransformation. Reducing Cr(VI) to Cr(III) is one example of such a process. These biological methods require no harsh chemicals, avoid secondary waste creation, and can be optimized using engineered microbial consortia to improve *in‐situ* performance and treatment efficiency.

## 3 Bacterial Chromium Reduction Mechanisms

Bacteria reduce Cr(VI) to Cr(III) through non‐enzymatic and enzymatic pathways involving diverse genes and metabolic processes. Non‐enzymatic reduction processes depend on bacterial metabolites or structural elements rather than direct enzyme catalysis. Organic acids, reduced sulfur compounds, and cellular thiols such as glutathione (GSH) can reduce Cr(VI) through spontaneous redox reactions (Sun et al. 2020; Singh et al. 2022). GSH reduces Cr(VI) to Cr(III) via multistep electron transfer, forming glutathione disulfide (GSSG) (Wiegand et al. 1984). Metallothioneins, a cysteine‐rich proteins, also contribute Cr(VI) reduction via their sulfhydryl groups (Krepkiy et al. 2003). Additionally, bacterial siderophores like catecholates and hydroxamates can mediate Cr(VI) reduction through electron‐donating functional groups (Yu et al. 2025; D. Zhang et al. 2022).

In the enzymatic pathway, bacterial enzymes that reduce Cr(VI) to Cr(III), commonly referred to as chromate reductases, are often encoded by the *chrR* gene. However, their primary physiological roles may extend beyond chromate reduction. For example, the chromate reductase from *Pseudomonas putida* (PsChrR), which utilizes either NADH or NADPH as electron donors, might be a reductase with different primary roles (Ishibashi et al. 1990; Park et al. 2000). The enzymatic mechanisms of Cr(VI) reduction can occur in two types, often described as Type I (“tight”) versus Type II (“semi‐tight”) reaction. In Type I, the enzyme channels electrons from NAD(P)H‐reduced flavin directly to enzyme‐bound Cr(VI)/Cr(V) intermediates, minimizing radical escape and lowering ROS. In Type II, the enzyme primarily reduces a diffusible mediator (e.g., FMNH₂/quinones), which then reduces Cr(VI) off‐enzyme, a looser coupling that permits more redox cycling and ROS (Thatoi et al. 2014). In *Stenotrophomonas maltophilia*, a ChrR‐like enzyme is also functional, and Cr(VI) exposure has been shown to induce a 25 kDa protein correlating with Cr(VI) reduction (Baldiris et al. 2018).

Nitroreductases, including members of the NfsA, NfsB, and NitR families, are flavin‐containing enzymes that typically reduce nitroaromatic compounds but can also act on Cr(VI) due to their broad substrate specificity (Kwak et al. 2003; Ramli et al. 2023). For instance, *Bacillus sp*. CRB‐1 upregulates the *nitR*2 gene under Cr(VI) stress (Zhu et al. 2019), whereas in *Bacillus cereus* SJ1, *nitR* genes are constitutively expressed (He et al. 2010a). Although they can reduce Cr(VI), these enzymes are generally less efficient than dedicated chromate reductases (Opperman et al. 2008).

Flavin reductases, which regenerate flavin cofactors, also play a role in Cr(VI) detoxification. In *E. coli*, the *yieF* gene encodes a soluble NADH/NADPH‐dependent oxidoreductase that acts as a four‐electron chromate reductase, generating less ROS and reducing oxidative stress through a type I reaction (Ackerley et al. 2004). Meanwhile, in *Staphylococcus aureus*, the *nfoR* gene encodes an NAD(P)H‐dependent FMN reductase in which the reduced flavin, rather than the enzyme itself, mediates Cr(VI) reduction (O'Neill et al. 2020).

Among the diverse microorganisms capable of interacting with chromium, species from the genera *Stenotrophomonas* (Gram‐negative) and *Bacillus* (Gram‐positive) are frequently isolated from chromium‐contaminated environments. Therefore, we will further focus on these two genera.

## 4 Hexavalent Chromium Reduction by *Stenotrophomonas* Species

Several studies have demonstrated *Stenotrophomonas* species as chromium‐reducing species (Table 1) with strains frequently isolated from chromium‐contaminated environments, reflecting the distribution and natural resistance of this genus to chromium. Some *Stenotrophomonas* strains, such as DSM14405 (Gao et al. 2020) and D6 (S. Ge et al. 2015), demonstrate complete Cr(VI) reduction, while several other strains, including NA2, D6, Crt94‐4A, and OS4, were capable of very high reduction even at elevated initial Cr(VI) concentration (Baldiris et al. 2018; Oves et al. 2013; Shreif et al. 2022).

**Table 1:** Reduction performance of Cr(VI) by *Stenotrophomonas* strains across reported conditions and key findings.

Species/Strain	Initial [Cr(VI)] (mg/L or mg/g)	Optimal pH	Optimal Temp (°C)	Time	Reduction/Removal (%)	Rate (mg/L/h)	Key Notes	Reference
*S. maltophilia* OS4	100 mg/L	7.0	35	48 h	91%	~1.90	High tolerance (1200 mg/L); likely enzymatic; dual use for AgNP synthesis.	Oves et al. (2013)
*S. maltophilia* SRS05	490 mg/L (in effluent)	7.0	35	15 days	46.9%	~0.64	Focus on bioadsorption in real tannery effluent; optimized with statistical methods.	Raman et al. (2018); Sundari (2017)
*Stenotrophomonas* sp. JD1	300 mg/L	Not Tested	30	90 h	30%	~1.00	Protective mechanisms: EPS production & biofilm formation; indirect extracellular reduction.	(Morel et al. (2009))
*Stenotrophomonas* sp. D6	200 mg/L (growing cells)	9.0	35	72 h	100%	~2.78	Extremely high tolerance (1,600 mg/L); permeabilized cells showed superior, reusable activity in real wastewater.	Ge et al. (2015)
*S. rhizophila* JC1	40 mg/L	7.0	35	24 h	≈0%	N/A	Important negative result. Effective for Pb/Cu but not Cr. Genomic analysis confirmed lack of Cr‐specific reduction genes.	S.‐C. Sun et al. (2021)
*S. rhizophila* PM6/PM7	5 mg/L (in LB medium)	Not Tested	30	48 h	81‐82%	~0.085	High efficiency at low conc. in rich media, but reduction was inhibited in presence of polypropylene (PP).	Denaro et al. (2025)
*Stenotrophomonas* sp. Crt94‐4A	88.5 mg/L (optimized)	7.0	37	72 h	96%	~1.18	Statistical optimization (RSM) significantly increased reduction efficiency. High tolerance (712 mg/L).	Shreif et al. (2022)
*S. maltophilia* ZA‐6	~26 mg/L (500 µM)	7.2	30	56 h	100%	~0.46	Intracellular enzymatic (NADH‐dependent) reduction confirmed; linked resistance to reduction.	Alam and Ahmad (2012)
*Stenotrophomonas* sp. (JQ707953)	16.59 mg/L (optimized)	7.38	~32	4.07 days	81.3%	~0.14	Simultaneous reduction with phenol as electron donor; optimized via statistical methods.	Dharmaraj and Muthukumar (2013)
*S. acidaminiphila* 4‐1	0.2 mg/g (in soil)	Not Tested	30	7 days	74.9%	N/A (soil)	Enhanced removal in soil using biochar immobilization (loofah best); converted Cr to more stable forms.	Wang et al. (2024)
*Stenotrophomonas* sp. D6 (Modified Medium)	100 mg/L	9.0	30	28 h	98.5%	~3.52	Medium modification (replacing NaCl with NH₄Cl/KH₂PO₄) drastically enhanced extracellular reductase secretion. Reusable lyophilized powder.	Zha et al. (2024)
*S. rhizophila* DSM14405T	50 mg/L	7.5	30	28 h	100%	~1.79	Transcriptomic analysis revealed resistance mechanisms (DNA repair, reduced sulfate uptake); PGPR strain, a safer alternative to *S. maltophilia*.	Gao et al. (2020)
*S. maltophilia* K8	4 mg/L	7.0	30	120 h	58%	~0.019	Simultaneous reduction with PCBs as electron donor; performance was inhibited by co‐contamination.	Yasir et al. (2021)
*S. acidaminiphila* 4‐1	15 mg/L	7.2	30	7 days	75.7%	~0.068	Multi‐step mechanism (EPS biosorption, reduction, intracellular accumulation); tested for co‐remediation with diesel.	Li et al. (2021)
*S. maltophilia* NA2	500 mg/L	7.0	37	72 h	92%	~6.39	Extreme tolerance (MIC ~ 7400 mg/L); ChrR gene identified; Cr(VI) induces a ~ 25 kDa soluble protein.	Baldiris et al. (2018)
*Stenotrophomonas* sp. SY1	5.2 mg/L (100 µM)	Not Tested	28	36 h	100%	~0.144	Novel mechanism via hemeprotein HhuH; links Cr(VI) reduction to iron starvation response. Also chelates Cd(II).	Zhou et al. (2025)

Among *Stenotrophomonas* species, *S. maltophilia* is most frequently isolated and consistently exhibits chromium‐reducing activity. However, its role as an opportunistic human pathogen and its antibiotic resistance limit its use for *in‐situ* applications. In contrast, recent studies have highlighted the potential of non‐pathogenic species such as *S. rhizophila* DSM14405 (Gao et al. 2020) and *S. acidaminiphila* 4‐1 (L. Li et al. 2021; X. Wang et al. 2024), both isolated from plant rhizospheres. These strains also exhibit plant‐growth‐promoting activity, making them attractive candidates for bioremediation coupled with revegetation.

The importance of distinguishing strain‐specific capability is underscored by Sun et. al (2021), who reported that *S. rhizophila* JC1, despite tolerating up to 40 mg/L of chromium, showed no detectable chromium‐reducing activity. This finding emphasizes that chromium reduction is not a universal trait within the *Stenotrophomonas* genus and cannot be inferred phylogenetically; functional screening is therefore essential.

Most chromium‐reducing *Stenotrophomonas* strains operate optimally at mesophilic temperatures (30°C–37°C) and neutral‐to‐slightly alkaline pH (7.0–7.5). An exception is *Stenotrophomonas* sp. D6 (S. Ge et al. 2015), which performs best at pH 9.0, suggesting its potential usefulness in treating alkaline industrial effluent where most microbes are less effective. Although most *Stenotrophomonas* strains perform Cr(VI) reduction optimally under near‐neutral conditions similar to ambient environments, many contaminated sites have pH values below 7.0. This limitation can be mitigated through soil neutralization (e.g., lime amendment) (Dharmaraj and Muthukumar 2013). Natural buffering by soil minerals, biofilm formation, which is common in *Stenotrophomonas* (Farooq et al. 2025; Morel et al. 2009), and other organic material in soil can also increase local pH, creating microenvironments suitable for Cr(VI) reduction. Moreover, *Stenotrophomonas* has also been reported to precipitate calcite (Enyedi et al. 2020), which can further raise environmental pH.

The impact of co‐contaminants represents another critical factor. Some contaminants, such as phenol (Dharmaraj and Muthukumar 2013) and diesel (L. Li et al. 2021), can act as electron donors that support or at least do not hinder Cr(VI) reduction. Other metals, such as Cd(II), can even induce chromium reductase activity in *Stenotrophomonas sp*. SY1 (Zhou et al. 2025). However, certain pollutants impose metabolic trade‐offs and inhibit reduction. For example, polypropylene, a common polymer used in plastic, substantially inhibited Cr(VI) reduction (Denaro et al. 2025). In *Stenotrophomonas sp*. TD3, simultaneous exposure to Cr(VI) and Zn(II) caused mutual inhibition, with Zn(II) suppressing Cr(VI) reduction and Cr(VI) drastically decreasing Zn(II) biosorption (S. Ge and Ge 2016). This antagonism likely reflects a metabolic shift toward processing competing substrates. These effects underscore that laboratory efficiencies might overestimate real‐world performance, highlighting the need to evaluate *Stenotrophomonas* under realistic, mixed‐contaminant scenarios.

**Figure 1:**
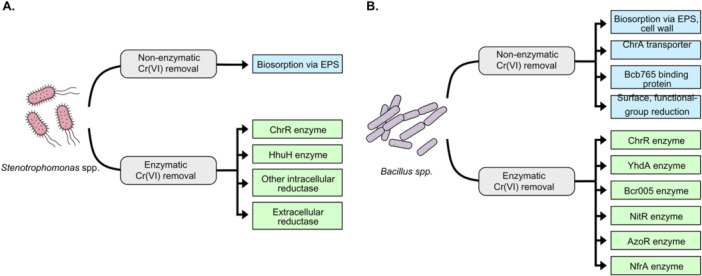
Schematic overview of non‐enzymatic and enzymatic Cr(VI) removal mechanisms in *Stenotrophomonas* (A) and *Bacillus* (B). Non‐enzymatic mechanisms include biosorption to extracellular polymeric substances (EPS) in both genera, with additional non‐enzymatic routes documented for *Bacillus*. Enzymatic mechanisms are mediated by diverse reductases that convert Cr(VI) to Cr(III). Mechanisms are non‐hierarchical and may act concurrently.

Understanding the metabolic basis of Cr(VI) reduction is essential to explain the variability in strain performance and to guide the development of more robust *Stenotrophomonas*‐based bioremediation strategies. Some species employ cellular mechanisms such as biosorption to sequester Cr(VI) with extracellular polymeric substance (EPS) on the cell surface (Morel et al. 2009; Sundari 2017), while in other species, Cr(VI) reduction occurs primarily through enzymatic processes (Figure 1A). Cellular fractionation studies have shown that this activity can be mediated by intracellular (cytosolic) enzymes, as in *Stenotrophomonas* ZA‐6 (Alam and Ahmad 2012) and NA2 (Baldiris et al. 2018), or by extracellular enzymes secreted into the medium, which can be significantly enhanced by modifying culture conditions, as demonstrated in *Stenotrophomonas sp*. D6 (Zha et al. 2024). These cellular and enzymatic processes are not mutually exclusive and may occur sequentially, as seen in *S. acidaminiphila* 4‐1, which performs initial biosorption followed by enzymatic reduction and intracellular accumulation of Cr(III) (L. Li et al. 2021).

The molecular basis of Cr(VI) resistance and reduction in *Stenotrophomonas* is gradually being revealed through advances in omics technologies. Baldiris et al. (2018) successfully amplified the chromate reductase gene *chrR* from *S. maltophilia* NA2, providing genetic evidence for a functional reductase. Interestingly, its expression appears strain‐dependent; it is inducible in strain NA2 but constitutive in *Stenotrophomonas sp. TD3* (S. Ge and Ge 2016), suggesting that some strains are constantly prepared for chromium detoxification. Whole‐genome sequencing has also been informative; Sun et al. (2021) confirmed the absence of known reductase genes in the non‐reductive *S. rhizophila* JC1, directly linking Cr(VI) reduction activity to the presence of reductase genes.

Transcriptomic analyses further illuminate bacterial responses to Cr(VI) stress. In *S. rhizophila* DSM14405, Cr(VI) exposure upregulated genes involved in DNA repair, oxidative stress defense, and iron homeostasis while downregulating sulfate transporter genes, likely to reduce chromate uptake due to its structural similarity to sulfate (Gao et al. 2020). More recently, proteomics analysis of *Stenotrophomonas* sp. SY1 revealed that Cr(VI) exposure induces an iron starvation response, leading to upregulation of the ferruginous hemeprotein HhuH, which both mediates Cr(VI) reduction and restores iron homeostasis (Zhou et al. 2025). Together, these omics studies depict a multi‐layered cellular defense system against Cr(VI), integrating specific reductases with broad stress response networks.

## 5 Hexavalent Chromium Reduction by *Bacillus* Species

*Bacillus* is a large and diverse genus of Gram‐positive bacteria. Members of the *Bacillus* genus have been frequently isolated from a wide range of chromium‐polluted and extreme environments (Table 2), including chromite mines (Dhal et al. 2010), alkaline soda lakes (Ibrahim et al. 2012), and tannery effluents (Elahi et al. 2022). Their ubiquity across such diverse and hostile habitats reflects ecological versatility and physiological robustness that enable survival under sustained oxidative and metal stress. This resilience largely stems from their ability to form endospores and active strong stress‐response systems, allowing persistence where most microorganisms perish (McKenney et al. 2013).

**Table 2:** Reduction performance of Cr(VI) by *Bacillus* strains across reported conditions and key findings.

Species/Strain	Initial [Cr(VI)] (mg/L or mg/kg)	Optimal pH	Optimal Temp (°C)	Time	Reduction/Removal (%)	Rate (mg/L/h)	Key Notes	Reference
*Bacillus cereus* b‐525k	104 mg/L	8.0	37	6 days	99%	~0.72	Extreme tolerance (MIC ~ 1664 mg/L); pilot‐scale test in real effluent; strong antioxidant response indicates intracellular reduction.	Elahi et al. (2022)
*Bacillus subtilis* SL‐44	100 mg/L	7.2	30	72 h	31.2%	~0.43	Baseline performance; reduction forms intracellular Cr₂O₃ nanoparticles.	T. Li et al. (2023)
*B. subtilis* SL‐44 (+ Humic Acid)	100 mg/L	7.2	30	72 h	39.8%	~0.55	Enhanced reduction using humic acid (HA) as an electron shuttle and protectant. Reduced Cr(VI) toxicity to cells. Effective in soil.	T. Li et al. (2023)
*Bacillus sp*. CRB‐1	50 mg/L	7.0–9.0	36–42	24 h	100%	~2.08	Identified *nitR2* gene as a novel NADPH‐dependent reductase for reduction and *chrA* gene for resistance. Final product was a soluble Cr(III)‐EPS complex.	Zhu et al. (2019)
*Bacillus paramycoides* Cr6	200 mg/L	8.0	31	18 h	100%	~11.11	Extreme tolerance (MIC 2500 mg/L) & rapid rate. Transcriptomics identified a novel dual intracellular mechanism: *bcr005* (reductase) and *bcb765* (binding protein) work in parallel.	Gu et al. (2023)
*Bacillus methylotrophicus*	Not specified (effluent)	7.0	30	48 h	91.3%	N/A	Focus on extracellular, inducible, GSH‐dependent chromate reductase. Crude enzyme was effective in real tannery effluent.	Sandana Mala et al. (2015)
*Bacillus subtilis* BDSA1	100 mg/L	7.0	37	24 h	40%	~1.67	Primarily a genome mining study. Identified *chrA* gene for Cr resistance. Moderate reduction but also removes Cadmium (43%). Predicted non‐pathogenic PGPR strain.	Saikat et al. (2024)
*Bacillus cereus* SJ1	52 mg/L	7.0	37	57 h	~97%	~0.88	High tolerance (MIC ~ 1560 mg/L); Genomic analysis identified inducible *chrA1* for resistance and constitutive *azoR*/*nitR* for reduction; evidence of HGT.	He et al. (2010b)
*Bacillus sp*. FY1	200 mg/L	8.0	30–35	24 h	78%	~6.50	High tolerance (1000 mg/L) and rapid reduction rate. Also demonstrated high removal efficiency (85%) in contaminated soil.	Xiao et al. (2017)
*Bacillus sp*. KSUCr9a	10.4 (200 µM)	9.0	35	48 h	100%	~0.22	Extreme tolerance (MIC ~ 4160 mg/L); alkaliphilic & halotolerant; reduction is optimal under static (low oxygen) conditions; reusable.	Ibrahim et al. (2012)
*Bacillus sp*. QC1‐2	~17 (0.33 mM)	7.0–7.5	30	22 h	~100%	~0.77	Foundational study; identified a soluble, intracellular, NADH‐dependent enzyme; confirmed final product was soluble Cr(III) that remained in the supernatant.	Campos et al. (1995)
*Bacillus sp*. MH778713	1474 mg/L	Not Tested	37	7 h	> 95% (at lower conc.)	N/A	Extreme hyper‐tolerance (MIC 15,000 mg/L); primary mechanism is biosorption/bioaccumulation (116 mg/g capacity). Assists plant phytoremediation.	Ramírez et al. (2019)
*Bacillus subtilis* MNU16	50 mg/L	Not Tested	30	72 h	75%	~0.52	PGPR strain with PGP traits confirmed. TEM analysis showed intracellular electron dense precipitates, indicating intracellular reduction.	Upadhyay et al. (2017)
*Bacillus subtilis* BYCr‐1	10.4 (0.2 mM)	6.0–7.0	37	48 h	~100%	~0.22	Identified and confirmed the role of the inducible *nfrA* gene in Cr(VI) reduction. TEM showed precipitates both inside and outside cells.	Zheng et al. (2015)
*Bacillus toyonensis* LBA36	30 mg/L	Not Tested (~7.2)	30	56 h	98%	~0.52	Primarily extracellular reduction (98%) versus adsorption (9%) confirmed by XPS/FTIR. PGPR strain that assists radish phytoremediation.	Tan et al. (2024)
*Bacillus sp*. FM1	100 mg/L	8.0	37	48 h	100%	~2.08	High tolerance (1000 mg/L); reduction enhanced by glucose; inhibited by co‐contaminant metals (Cd, Zn, Cu).	Masood and Malik (2011)
*Bacillus cereus* IST105	968 mg/L (in effluent)	7.0	30	72 h	> 76%	~10.21	Mechanism is biosorption/accumulation in real effluent. Detoxification confirmed via human cell line (MTT) assay.	Naik et al. (2012)
*Bacillus thuringiensis* V45	50 mg/L	7.0	35	96 h	86.4%	~0.45	High tolerance (520 mg/L); Mechanism is biosorption/bioreduction via surface functional groups.	Suresh et al. (2021)
*B. paramycoides* S48 (Whole Cell)	500 mg/L	7.0	35	96 h	~65%	~3.39	High tolerance (MIC 1500 mg/L); baseline performance of native strain before enzyme cloning.	Kalsoom et al. (2025)
*B. paramycoides* S48 (Recombinant Enzyme)	500 mg/L	7.0	35	96 h	100%	~5.21	Recombinant chromate reductase (BparChR) expressed in *E. coli* showed significantly enhanced reduction compared to whole cells.	Kalsoom et al. (2025)
*Bacillus wiedmannii* SA1	200 mg/L	8.0	35	24 h	70.3%	~5.86	Extracellular enzymatic reduction driven by glucose. Genes for azoreductase and ChrR were identified.	Adhikary et al. (2025)
*Bacillus sp*. CSB‐4	100 mg/L	7.0	35	144 h	> 90%	~0.63	High tolerance (2000 mg/L); surface reduction leads to precipitation of chromium hydrogen phosphate in phosphate‐containing medium.	Dhal et al. (2010)
*Bacillus cereus* ZY‐2009	20 mg/L	7.0	30	48 h	81.2%	~0.34	Extracellular fractions showed highest activity; reduction strongly stimulated by Fe³⁺ and Cu²⁺.	Y. Sun et al. (2023)

Several *Bacillus* strains exhibit remarkable tolerance to Cr(VI), with MICs typically ranging from 500 to 2500 mg/L. Notably, *B. paramycoides* Cr6 tolerates up to 2500 mg/L and completely reduces 200 mg/L Cr(VI) within 18 h, achieving an exceptional rate of ~11.11 mg/L·h⁻¹ (Gu et al. 2023). Similarly, *B. cereus* b‐525k tolerates up to 1664 mg/L and achieves 99% reduction within 6 days (Elahi et al. 2022), while the hyper‐tolerant *Bacillus* sp. MH778713 can survive concentrations as high as 15,000 mg/L, relying primarily on bioaccumulation and biosorption to mitigate toxicity (Ramírez et al. 2019). Such traits highlight the genus strong potential as a bioremediation agent in high‐load or industrial effluents where conventional microbial consortia may not survive. Cr(VI) detoxification by *Bacillus* species occurs through multiple mechanisms, including intracellular and extracellular enzymatic reduction, as well as surface‐associated biosorption or biomineralization (Figure 1B).

Among these mechanisms, intracellular enzymatic reduction is the most common pathway. The first reduction activity was characterized in *Bacillus* sp. QC1‐2, where a soluble NADH‐dependent chromate reductase converted Cr(VI) to soluble Cr(III) complexes (Campos et al. 1995). Subsequent studies have identified a range of reductases belonging to flavin‐dependent and nitroreductase families. One of the best characterized example is the YhdA, an NADPH‐dependent flavin mononucleotide (FMN) oxidoreductase from *B. subtilis* that also exhibits azoreductase activity (Valenzuela‐García et al. 2020). YhdA forms a tetrameric complex homologous to known bacterial chromate reductases such as ChrR (*Pseudomonas putida*) and YieF (*Escherichia coli*), sharing conserved FMN‐ and NAD(P)H‐binding motifs. Overexpression of YhdA enhances Cr(VI) tolerance and reduces reactive oxygen species (ROS) accumulation, suggesting a multi‐electron reduction mechanism that efficiently transfers electrons to Cr(VI) while minimizing ROS generation (Valenzuela‐García et al. 2020). Although its catalytic mechanism remains unresolved, YdhA may operate through a semi‐tight two‐electron transfer process similar to ChrR, which generates more ROS, or through a tighter coupling akin to YieF. These intracellular reduction mechanisms are environmentally relevant, as they help explain the strong redox resilience of *Bacillus* cells, consistent with the pronounced antioxidant responses observed in *B. cereus* b‐525k during intracellular Cr(VI) reduction (Elahi et al. 2022).

Transmission electron microscopy (TEM) and biochemical assays further support intracellular Cr(VI) reduction as a major pathway. Strains such as *B. subtilis* SL‐44 and *B. subtilis* MNU16 form electron‐dense Cr(III) nanoparticles within the cytoplasm following exposure (T. Li et al. 2023; Upadhyay et al. 2017). The addition of humic acid as an electron shuttle increased the reduction efficiency of SL‐44 from 31.2% to 39.8%, demonstrating how exogenous redox mediators can enhance biotransformation rates by facilitating extracellular electron transfer (T. Li et al. 2023). Similar mechanisms have been reported in *Bacillus* sp. CRB‐1, where a novel NADPH‐dependent reductase (*nitR2*) and an efflux pump (*chrA*) mediate complete reduction of 50 mg/L Cr(VI) within 24 h (Zhu et al. 2019). Likewise, transcriptomic analysis of *B. paramycoides* Cr6 identified two unique genes, *bcr005* (chromate reductase) and *bcb765* (Cr(VI)‐binding protein), that function cooperatively in parallel intracellular pathways (Gu et al. 2023). The coexistence of a catalytic reductase and a Cr(VI)‐binding protein illustrates how *Bacillus* species couple enzymatic detoxification with intracellular metal sequestration for efficient Cr(VI) management.

Genomic and proteomic studies have increasingly clarified the genetic basis of Cr(VI) resistance and reduction in *Bacillus*. The chromate efflux pump encoded by *chrA* remains the most consistently observed resistance determinant, reported in *B. subtilis* BDSA1 (Saikat et al. 2024) and *B. cereus* SJ1 (He et al. 2010b). In contrast, Cr(VI) reduction is mediated by a diverse set of reductases, including NAD(P)H‐dependent flavin reductases (YhdA), nitroreductases (NitR), and FMN‐dependent oxidoreductases (NfrA). The role of NfrA was experimentally verified in *B. subtilis* BYCr‐1, where its induction enabled near‐complete reduction of 10.4 mg/L Cr(VI) (Zheng et al. 2015). Furthermore, recombinant expression of *BparChR* from *B. paramycoides* S48 in *E. coli* improved Cr(VI) reduction efficiency from 65% (whole cell) to 100%, confirming that chromate reductases can be effectively transferred and functionally expressed across heterologous hosts (Kalsoom et al. 2025). Together, these findings indicate that Cr(VI) detoxification in *Bacillus* involves a modular genetic and physiological system, where resistance and reduction are independently regulated yet synergistically under stress. The integration of multi‐pathway reduction, efflux, and biosorption mechanisms highlights the remarkable adaptability of *Bacillus* species and explains their prevalence in Cr(VI)‐rich environments, making them compelling candidates for bioaugmentation and enzyme‐based remediation technologies.

## 6 Application of *Stenotrophomonas* and *Bacillus* in Chromium Bioremediations

Comparing Cr(VI) reduction strategies from *Stenotrophomonas* and *Bacillus* reveals both overlapping mechanisms and distinct approaches. *Stenotrophomonas* species, particularly *S. maltophilia* D6 and NA2, demonstrate high Cr(VI) reduction efficiency, up to 98%–100% reduction, under neutral to alkaline conditions (S. Ge et al. 2015; Zha et al. 2024) and extreme tolerance to chromium (MIC > 7400 mg/L; Baldiris et al. 2018). Their metabolic versatility extends to the degradation of co‐contaminants such as phenol, diesel, and other organics, supported by extracellular enzymatic activity and electron shuttling. However, this genus primarily relies on intracellular enzymatic reduction mediated by NADH‐dependent reductases such as ChrR or hemeproteins like HhuH that link chromium detoxification with iron homeostasis (Zhou et al. 2025). These cytoplasmic mechanisms are efficient but risk intracellular exposure to Cr(VI) and reactive intermediates (Cr(V) species), increasing oxidative stress.

In contrast, members of the *Bacillus* genus exhibit broader and more diverse Cr(VI) removal mechanisms (Figure 1B), combining intracellular reduction, extracellular enzymatic activity, and biosorption. *B. paramycoides* Cr6 exemplifies rapid intracellular reduction through parallel pathways involving both chromate reductase (Bcr005) and Cr(VI)‐binding protein (Bcb765), achieving complete reduction of 200 mg/L within 18 h at an approximate rate of ~11.11 mg/L·h⁻¹ (Gu et al. 2023). Meanwhile, *B. cereus* b‐525k and *Bacillus sp*. MH778713 exhibit remarkable tolerance (MIC > 1500–15,000 mg/L) via antioxidant defense and biosorption mechanisms (Elahi et al. 2022; Ramírez et al. 2019)). Several *Bacillus* species, such as *B. toyonensis* LBA36 and *B. subtilis* MNU16, also function as plant growth–promoting rhizobacteria (PGPR), facilitating simultaneous detoxification and plant establishment in contaminated soils (A. Tan et al. 2024; Upadhyay et al. 2017). Their spore‐forming ability and environmental resilience make them exceptionally well‐suited for fluctuating soil and effluent environments.

A growing body of research demonstrates that microbial consortia consistently outperform single strains in chromium bioreduction, owing to functional diversity, metabolic cross‐feeding, and improved stress tolerance (Panneerselvam et al. 2013). Consortia derived from contaminated matrices, such as industrial sludge and tannery waste, frequently exhibit high reduction capacities (Desai et al. 2009; Panneerselvam et al. 2013). For example, a *Bacillus altitudinis–B. tropicus* consortium reached 92% total chromium removal under alkaline pH 9 within 48 h, while proteomic analysis confirmed enzymatic reduction coupled with Cr(III) bioaccumulation (Hamed et al. 2025). Another study reported complete removal of 50 mg/L Cr(VI) within 5 h by a sludge‐enriched community and sustained performance up to 300 mg/L (Kholisa et al. 2021). These systems generally perform optimally near neutral pH (6.5–7.5) and mesophilic temperatures (~30°C–37°C), conditions compatible with both *Bacillus* and *Stenotrophomonas* activity (Desai et al. 2009; Kholisa et al. 2021; Piñón‐Castillo et al. 2010). Division of labor is a central feature of consortial efficiency (Desai et al. 2009; Larik et al. 2016). In mixed cultures, certain members specialize in enzymatic Cr(VI) reduction via reductases and dehydrogenases, while others provide extracellular polymeric substances (EPS) that sequester or immobilize Cr(VI), minimizing cytotoxic exposure. This cooperative behavior mirrors the potential synergy between *Bacillus* and *Stenotrophomonas*: both can rapidly detoxify soluble Cr(VI) fractions through extracellular or periplasmic reductases (Morel et al. 2009; Zha et al. 2024; Adhikary et al. 2025), while *Bacillus* performs intracellular reduction and long‐term immobilization through biosorption or formation of stable Cr(III)–EPS complexes (Zhu et al. 2019).

Such dual consortia have clear advantages under environmental stress. In mixed‐contaminant systems (e.g., Cr(VI) with dyes or hydrocarbons), *Bacillus–Stenotrophomonas* associations sustain high Cr(VI) reduction while co‐degrading secondary pollutants (Desai et al. 2009; Larik et al. 2016). These traits also translate effectively to continuous‐flow bioreactors, where mixed consortia maintain high removal efficiency at steady state (Desai et al. 2009). Beyond direct detoxification, *Bacillus–Stenotrophomonas* consortia show promise in rhizo‐assisted systems, where *Bacillus* supports plant growth (Saikat et al. 2024; Upadhyay et al. 2017) and *Stenotrophomonas* contributes metal reduction in the rhizosphere (L. Li et al. 2021; X. Wang et al. 2024). These findings highlight a realistic pathway for scalable bioremediation: combining spore‐forming *Bacillus* for persistence with metabolically versatile *Stenotrophomonas* for rapid detoxification. While *Bacillus–Stenotrophomonas* partnerships are particularly synergistic, broader consortia incorporating bacteria from genus *Pseudomonas*, *Enterobacter*, *Arthrobacter*, or *Halomonas* have also demonstrated enhanced Cr(VI) removal under multi‐pollutant or high‐salinity conditions (Kholisa et al. 2021; Panneerselvam et al. 2013; Piñón‐Castillo et al. 2010). These communities expand electron donor versatility and pollutant range but require careful balance to prevent dominance by fast‐growing species. Mixed consortia thus represent an adaptive, multi‐functional framework where metabolic redundancy ensures stability across fluctuating field environments.

Pathogenicity concerns, especially for *S. maltophilia*, remain a major deterrent for its use in open environmental systems or applications where human exposure is possible (Brooke 2012). However, the risk associated with its implementation can be effectively managed by following a cautious approach by prioritizing *ex‐situ* solutions such as treatment in contained bioreactor systems which limit environmental release and improve operational control over *in‐situ* implementation. Several studies have reported degradation of dyes in industrial effluent (Galai et al. 2009; Mishra et al. 2024), TNT (Lee et al. 2002), and herbicides (Galíndez‐Nájera et al. 2011) using *Stenotrophomonas* species in bioreactor settings with free or immobilized cells. Nonetheless, ex‐situ treatment can be resource‐intensive and limit scalability for large contaminated areas, which offsets the advantages of bioremediation that stem from the low cost and scalability of *in‐situ* applications. In this context, non‐pathogenic *Stenotrophomonas* species such as *S. rhizophila* DSM14405 (Gao et al. 2020) and *S. acidaminiphila* 4‐1 (L. Li et al. 2021; X. Wang et al. 2024), both capable of Cr(VI) reduction and plant association, represent safer alternatives for environmental deployment, particularly when paired with resilient *Bacillus* strains in controlled or semi‐natural consortia. In summary, microbial consortia anchored by *Bacillus* and *Stenotrophomonas* species integrate mechanistic complementarity, ecological robustness, and scalable performance. Their cooperative interaction, balancing extracellular detoxification, intracellular reduction represents a promising framework for next‐generation chromium bioremediation systems that bridge laboratory efficiency and field resilience.

## 7 Future Perspectives

Bioremediation is frequently portrayed as a better alternative to conventional environmental remediation methods due to several key advantages, such as environmental friendliness, cost‐effectiveness, waste reduction, and sustainability. Biological processes, be it from bacteria or plant activities, are inherently greener as they happen in natural settings under ambient conditions without harsh chemicals required in conventional remediation. This significantly reduced toxic waste, minimizing secondary pollution risks (Singh et al. 2023). Bioremediation, particularly *in‐situ* approaches or those where readily available biomass is utilized, is often considered more economical due to lower energy inputs to process the primary waste and secondary waste compared to processes like reverse osmosis or electrochemical treatments (Beretta et al. 2019). An analysis of water adsorbent material made out of organic material was found to be excellent for Cr(VI) removal. All of this leads to bioremediation most significant advantage: sustainability. By harnessing natural processes and renewable resources, bioremediation can be sustained without much environmental damage and aligns well with sustainability goals (Ayele and Godeto 2021).

However, despite the compelling advantages, implementation of bioremediation for Cr(VI) faces several challenges. One such challenge is the scalability and translation from laboratory scale to contaminated sites while still being practical and reliable (Acharyya et al. 2023). Contaminated sites often found in mining or industrial areas are highly heterogenous with various biogeochemistry and co‐contaminants, making it difficult to maintain the optimal condition easily achieved in lab settings (Sharma et al. 2022), which can affect the survival, activity, and effective distribution of microbial inoculants. Introducing exogenous microbes in a non‐native environment can also face challenges from competition with native microbial populations (Oro et al. 2024). However, this can be partially solved by identifying native bacteria with chromium reduction capability. Biological processes are also significantly slower than conventional chemical or physical methods, and their efficiency is also very sensitive to environmental factors like pH, temperature, redox potential and nutrient availability (Carlos et al. 2016). Another challenge is long‐term stability and the potential of re‐oxidation of Cr(III), which is not thermodynamically stable under oxidizing conditions and can be oxidized back into the toxic Cr(VI) (X. Tan et al. 2024). Cr(VI) can also be present in low bioavailability, which limits microbial or plants uptake (Sharma et al. 2022).

Due to this challenges, significant advancements must be made to address the limitations and further harness the bioremediation potential of *Bacillus* and *Stenotrophomonas* species. Future research should cover systems‐level understanding of chromium reduction in bacteria to cellular‐level engineering. Elucidating the regulatory networks of genes, electron‐transfer pathway, and stress‐response networks involved in Cr(VI) resistance and reduction in these bacteria through omics approaches could be beneficial in understanding the triggers for constitutive and enhanced reduction systems. This knowledge is crucial for investigating the synergistic or antagonistic effects within microbial consortia containing *Stenotrophomonas* and/or *Bacillus* species to design a more robust and resilient bioremediation system that can survive across wider pH and temperature range to improve survivability in real‐world contaminated sites. Optimizing nutrients for the bacteria and improving electron donor strategies can also improve bioremediation potential (Galani et al. 2022). Furthermore, integrating microbial consortia alongside a chromium hyperaccumulator plant to accelerate chromium removal and increase bioremediation potential (C. Wang et al. 2020) or designing a system that integrates conventional and bioremediation methods, such as an adsorption‐bioreduction system, can combine the strengths of biological processes with conventional methods (Acharyya et al. 2023) to create more resilient and effective remediation solutions.

At the molecular level, further enhancements might come from using purified, stable enzymes like *B. subtilis* YhdA for cell‐free systems (Valenzuela‐García et al. 2020) to protein engineering using genome‐editing techniques like CRISPR‐Cas9. Genome‐editing techniques open up a vast potential by genetically engineering non‐pathogenic microorganisms to precisely overexpress key reductase enzymes or produce enzymes with enhanced reduction, stability, and tolerance capabilities. Heterologous expression of chromate reductase gene has been demonstrated in several studies (Kalsoom et al. 2025; Sandana Mala et al. 2015) showing that genetic engineering techniques is invaluable in bioremediation efforts.

Biosafety concerns that limit the use of potent strains like *S. maltophilia* to *ex‐situ* systems, as previously discussed, can also be addressed using genome editing and synthetic biology tools to develop microbes that are safe for *in situ* use. Future strategies can focus on attenuating the pathogenic potential by precisely knocking out or deleting pathogenic genes, rendering them harmless while still able to perform their valuable remediation functions. One study has successfully constructed a CRISPR Interference system for *S. maltophilia* AGS‐1 to knock down *xanA* and *rpfF* genes, which play important roles in biofilm formation (Liu et al. 2023). Designing synthetic auxotrophy and kill‐switch circuit can also be used to confine bacteria with programmable self‐destruct upon exiting a defined environment (Gallagher et al. 2015; Hayashi et al. 2024; Rottinghaus et al. 2022). These kill‐switch systems have been demonstrated for *E. coli* (Rottinghaus et. al 2022) and *P. putida* (Asin‐Garcia et al. 2024), both of which also harbor *ChrR* gene (Eswaramoorthy et al. 2012; Gonzalez et al. 2005). The kill‐switch can substantially reduce the risk of the bacteria persisting or spreading outside the defined zone, in this case, a contaminated site. However, genetic containment still presents risks such as mutation and horizontal gene transfers, which could bypass engineered safeguards (Rottinghaus et. al 2022; Hayashi et. al 2024). Therefore, a pilot‐scale trial with robust risk assessments and monitoring for horizontal gene transfer, persistence of the kill switch, and effects on native microbial communities paired with physical containment alongside redundant safeguards and other criteria that follow regulatory frameworks on genetically‐engineered organisms, is needed before large‐scale deployment. A combined strategy using omics‐guided target discovery and consortium design, enzyme‐based solutions, and applications of bacterial genome editing with multi‐layer containment offers a balanced and safe path forward. These approaches can combine efficacy and biosafety, enabling scalable *in‐situ* chromium bioremediation.

## 8 Conclusion

Cr(VI) poses a significant and persistent threat to environmental quality and public health due to its toxicity, mobility, and widespread industrial use. Remediation is urgently needed. Conventional remediation methods offer established routes for Cr(VI) removal, but are often burdened by toxic secondary waste and high costs. Bioremediation using microorganisms and plants offers advantages in environmental friendliness, potential cost‐effectiveness, and reduced secondary waste generation. Several organisms, such as bacteria from the genus *Bacillus* and *Stenotrophomonas*, have shown high potential in laboratory and pilot studies. However, challenges such as sensitivity of biological process to highly variable conditions often found in contaminated sites, pathogenicity concerns for some *Stenotrophomonas species*, and ensuring long‐term stability of the remediated chromium before being re‐oxidized back to Cr(VI) hinder widespread field application and make scaling up from controlled environments difficult. Future progress in bioremediation of Cr(VI) likely lies in optimizing and integrating approaches by enhancing the robustness and efficiency of bioremediation agents and the potential of microbial consortia while ensuring the long‐term stability of Cr(III) through controlled mineralization or geochemical treatment.

## Author Contributions

**Ahmad Fadhlullah Husaini:** writing, editing. **Margaretta Christita:** conceptualization, supervision, review, editing, funding acquisition. **Arida Susilowati:** conceptualization (supporting), funding acquisition. **Rizal Maarif Rukmana:** writing. **Keni Vidilaseris:** supervision, review, editing, funding acquisition.

## Ethics Statement

The authors have nothing to report.

## Conflicts of Interest

The authors declare no conflicts of interest.
